# Hepatoprotective effect of Xiayuxue decoction ethyl acetate fraction against carbon tetrachloride-induced liver fibrosis in mice via inducing apoptosis and suppressing activation of hepatic stellate cells

**DOI:** 10.1080/13880209.2020.1855212

**Published:** 2020-12-17

**Authors:** Dingqi Zhang, Lijun Zhang, Gaofeng Chen, Ying Xu, Hailin Yang, Zhun Xiao, Jiamei Chen, Yongping Mu, Hua Zhang, Wei Liu, Ping Liu

**Affiliations:** a Key Laboratory of Liver and Kidney Diseases (Ministry of Education), Institute of Liver Diseases, Shuguang Hospital Affiliated to Shanghai University of Traditional Chinese Medicine, Shanghai, China; b Shanghai Key Laboratory of Traditional Chinese Clinical Medicine, Shanghai, China; c Institute of Interdisciplinary Complex Research, Shanghai University of Traditional Chinese Medicine, Shanghai, China

**Keywords:** Xiayuxue decoction, liver fibrosis, hepatic stellate cells, activation, apoptosis

## Abstract

**Context:**

Xiayuxue decoction (XYXD), a traditional Chinese medicine, is used for treating liver disease. However, the potential active constituents and mechanisms are still unclear.

**Objective:**

To explore the main active fraction extracts, active ingredients and possible mechanisms of XYXD for anti-hepatic fibrosis.

**Materials and methods:**

Different fractions including ethyl acetate fraction (EF) were prepared from XYXD. These fractions, especially EF, were used to evaluate cell viability, proliferation, cell cycle, cytotoxicity and activation in hepatic stellate cells (HSCs). Liver fibrosis model was established by CCl_4_ in C57BL/6 mice, and allocated to CCl_4_ group, XYXD group and EF group with normal mice as control. Further, mitochondrial apoptosis-related proteins of HSCs, destruction and angiogenesis of liver sinusoidal endothelial cells (LSECs) and active ingredients of EF were evaluated.

**Results:**

The inhibition of proliferation, increase of S or/and G2/M phase population and suppression of α-SMA and COL-1 expression were obeserved in EF treated-JS1 and -LX2. Liver fibrosis-related indicators were improved by EF similar to XYXD *in vivo*. EF induced the apoptosis of HSCs in CCl_4_-induced fibrosis, and inhibited the expression of HSCs apoptosis pathway-related proteins (JNK and p38-MAPKs), and LSECs destruction and angiogenesis. Multiple ingredients (emodin, rhein, aloe-emodin, prunasin) in EF have shown inhibited the activation of JS1.

**Discussion and Conclusion:**

EF was the main active fraction extracts of XYXD, and the underlying mechanisms might relate to induction of HSCs apoptosis. Emodin, rhein, aloe-emodin and prunasin were main active ingredients of EF, which provides a potential drug for the treatment of liver fibrosis.

## Introduction

Hepatic fibrosis, the excessive deposition of extracellular matrix (ECM) proteins such as collagens, is caused by chronic liver diseases associated with various pathological factors including chronic viral hepatitis, alcoholic liver disease, and non-alcoholic steatohepatitis (Tsuchida and Friedman 2017). If left untreated, hepatic fibrosis may progress to liver cirrhosis, and ultimately develop into hepatocellular carcinoma (Schuppan and Afdhal 2008; Ellis and Mann 2012). Thus, alleviating liver injury and decreasing the progress of liver damage to fibrosis or even cancer is urgent. Hepatic stellate cells (HSCs) play a crucial role during the initiation and progression of hepatic fibrosis (Mederacke et al. 2013). In normal liver, HSCs stay in non­proliferative and quiescent state. Following liver injury, HSCs can be activated via cytokine stimulation of autocrine or paracrine including transforming growth factor-β (TGF-β), platelet-derived growth factor (PDGF), vascular endothelial growth factor (VEGF), and connective tissue growth factor (CTGF) (Friedman 2003; Chaudhary et al. 2007; Lee and Friedman 2011; Tsuchida and Friedman 2017). In this activation process, quiescent HSCs transform into fibrogenic, proliferative, contractile, and proinflammatory myofibroblast-like cells, which can produce ECM proteins (Tsuchida and Friedman 2017). Therefore, the elimination of the action of activated HSCs such as promoting its apoptosis, has become a significant strategy for antifibrotic therapies (Bataller and Brenner 2005). In addition, liver sinusoidal endothelial cells (LSECs), a highly specialised endothelial cell and a distinct cell with non-diaphragm fenestration organised in sieve plate, play important roles in fibrotic process (Poisson et al. 2017). In general, activated HSCs cause the loss of fenestration and pathological changes of LSECs, in which VEGF induced by hypoxia in liver tissue is an important factor. In this process, CD31, a marker of LSECs dedifferentiation, usually increases expression in LSECs (Thabut and Shah 2010). Differentiated LSECs can block HSCs activation and facilitate reversion to quiescence, but capillarized LSECs can positively accelerate HSCs activation (DeLeve 2015). Therefore, suppression of sinusoid capillarization is also an effective therapy for liver fibrosis.

In recent years, traditional Chinese medicine (TCM) has become popular for use in the treatment of many liver diseases, such as fibrosis (Zhou et al. 2014; Ma et al. 2017). Xiayuxue decoction (XYXD), a famous traditional Chinese herbal formulation consists of *Rheum palmatum* Linn (Polygonaceae), *Prunus persica* (Linn) Batsch (Rosaceae), and *Eupolyphaga sinensis* Walker (Corydiidae) was first found in Synopsis of the Golden Chamber in 200 AD. XYXD is a classic formulation for promoting blood circulation and removing blood stasis in science of TCM formulas, and has been used to treat puerpera-related abdominal pain and oligomenorrhea result from blood stasis (Liu et al. 2015). Based on the clinical practice of traditional Chinese medicine, the core pathogenesis and the main syndrome pathogenesis of liver fibrosis and cirrhosis after hepatitis were the consensus of blood stasis, qi and yin deficiency, damp heat intrinsic (Lv et al. 2012; Chi et al. 2016). XYXD is often used in the treatment of liver fbrosis and cirrhosis in China (Chen 2013) and is reported to attenuate liver fibrosis and/or cirrhosis mediated by carbon tetrachloride (CCl_4_) (Zhang et al. 2014; Ma et al. 2017), thioacetamide (Ding et al. 2012), or pig serum (Chen et al. 2012). Furthermore, XYXD has been also reported to play a protective effect in renal injury induced by bile duct ligation (Liu et al. 2015). In our previous studies confirmed that XYXD provides prominently anti-liver fibrotic effects in CCl_4_-induced rodents, which attenuated HSCs activation, collagen deposition, and LSECs defenestration (Zhang et al. 2014; Ma et al. 2017). However, the potential anti-liver fibrosis active constituents and XYXD possible mechanisms are still unclear.

This study investigates the main active fraction extracts of XYXD, its potential mechanisms and active ingredients for hepatic fibrosis. Three different fraction extracts of XYXD were first evaluated using *in vitro* experiments. Then, XYXD and its ethyl acetate extract (EF) were assessed *in vivo* experiments. Meanwhile, the chemical analysis of XYXD extract and its fractions were analysed using ultra-high-performance liquid chromatography-Q exactive hybrid quadrupole orbitrap high-resolution accurate mass spectrometriy (UHPLC-Q-Orbitrap HRMS). Further, the potential mechanisms of EF antihepatic fibrosis were studies with *in vivo* and *in vitro* experiments. Finally, the potential active ingredients of EF anti-liver fibrosis were examined via *in vitro* experiments.

## Materials and methods

### Chemicals and reagents

The *R. palmatum* (batch number: 180223), *P. persica* Batsch (batch number: 180202), and *E. sinensis* (batch number: 180116) were purchased from Shanghai Kangqiao Chinese Medicine Tablet Co., Ltd. (Shanghai, China) and authenticated by associate professor Wei Liu, Institute of Liver Diseases, Shanghai University of Traditional Chinese Medicine. Amygdalin (Amy, **1**), aloe-emodin (Alo, **9**), rhein (Rhe, **10**), emodin (Emo, **11**), chrysophanol (Chr, **12**) and physcion (Phy, **13**) were purchased from Dalian Meilun Biotechnology Co., Ltd. (Dalian, China). Aloe-emodin-8-*O*-β-d-glucopyranoside (A-8-G, **3**), rhein-8-*O*-β-d-glucopyranoside (R-8-G, **4**), emodin-1-*O*-glucoside (E-1-G, **5**), emodin-8-glucoside (E-8-G, **6**), chrysophanol-8-*O*-β-d-glucopyranoside (C-8-G, **7**) and chrysophanol-1-*O*-β-d-glucopyranoside (C-1-G, **8**) were purchased from Chengdu Biopurify Phytochemicals Ltd. (Chengdu, China). Prunasin (Pru, **2**) was obtained from Yuanye Biotech Company (Shanghai, China).

Aspertate aminotransferase (AST) test kit (Lot: 01AST180109) and alanine aminotransferase (ALT) test kit (Lot: 01AST180108) were purchased from Shanghai Huachen Bearing Technology Co., Ltd. (Shanghai, China). Total bilirubin (TBil) test kit (Lot: 180824101) was purchased from Medical System Biotechnology Co., Ltd. (Ningbo, China). Hydroxyproline (Hyp) assay kit (batch number: A030-2), Haematoxylin and Eosin (H&E) staining kit (batch number: 20180720) were purchased from Nanjing Jiancheng Bioengineering Institute (Nanjing, China). Trizol Reagents (#B900044-1000) was purchased from Shanghai Sangon Biological Engineering Co. Ltd. (Shanghai, China). Reverse transcriptase Assay kit (Lot: 831300) was obtained from Toyobo Co., Ltd. (Tokyo, Japan). Real-time reverse transcription polymerase chain reaction (RT-PCR) primers were designed and synthesised by Shanghai Sangon Biological Engineering Co. Ltd. (Shanghai, China). Cell counting kit-8 (CCK-8, Lot: LK814) was purchased from Dojindo Laboratories Co., Ltd. (Kumamoto, Japan), RIPA lysis buffer (Lot: p00133B), Lactate dehydrogenase (LDH) cytotoxicity assay kit (batch number: C0017), BeyoClick™ EdU Cell Proliferation Kit with Alexa Fluor 594 (Cat: C0078S) and Cell Cycle and Apoptosis Analysis kit (Cat: C1052) were obtained from Beyotimebiology Co., Ltd. (Shanghai, China). PVDF membrane was obtained from Millipore Corp. (Bedford, MA, USA). Recombinant Human TGF-β1 (Cat: 240-B) was obtained from R&D Systems, Inc. (Minneapolis, MN, USA). Antibodies against platelet endothelial cell adhesion molecule-1 (CD31, Cat: Ab28364), VEGF, Desmin (Cat: ab185033), α-smooth muscle actin (α-SMA, Cat: Ab5694), Bax (Cat: ab32503), Bcl-2 (Cat: ab141523), cytochrome C (Cat: ab13847), cleaved-caspase-3 (Cat: ab32042), caspase 3 (Cat: ab13847), p-p38 (Cat: ab4822), p-38 (Cat: ab31828) were obtained from Abcam, Inc. (Cambridge, UK). Type I collagen (COL-1, Cat: 234167) was purchased from Calbiochem, Inc. (San Diego, CA, USA). p-ERK (#4370), p-JNK (#4671), JNK (#9258), SB431542 (#14775), PD98059 (#9900), SB203580 (#5633), SP600125 (#8177) were purchased from Cell Signalling Technology, Inc. (MA, USA). ERK1/2 (Lot: 16443-1-AP), mouse monoclonal antibody against GAPDH (Lot: 60004-1-1 g) were obtained from ProteinTech Group Inc. (Chicago, IL, USA). Immortalised mice HSCs line JS1 cells and human HSCs line LX2 cells were purchased from Chinese Academy of Sciences Cell Bank (Shanghai, China). An immortalised human hepatocyte line L02 was obtained from Professor Hu-yiyang, Institute of Liver Diseases, Shanghai University of Traditional Chinese Medicine. Primary human hepatic sinusoidal endothelial cells (HHSECs) were purchased from Shanghai Wuwei biotechnology Co. Ltd. (Shanghai, China).

### Preparation and chemical analysis of XYXD and its fractions

XYXD was formulated by mixing three crude herbs including *R. palmatum*, *P. persica*, and *E. sinensis*, with daily dosage of 5, 5, and 3 g, respectively. All herbs (1300 g, including 500 g *R. palmatum*, 500 g *P. persica* and 300 g *E. sinensis*) were powdered and further extracted by 10 L 20% ethanol twice. Afterward, the extracted liquid was filtered and was subsequently evaporated through rotary vaporation under decompressing, and was vacuum dehydrated to afford extract of XYXD (758 g, extract yield was 58.31% from XYXD). Furthermore, a certain amount of extract of XYXD (310 g) was redissolved in 1 L water, and was followed by extraction with 10 volumes of petroleum ether and ethyl acetate five times, respectively. Finally, three different fractions (petroleum ether, ethyl acetate, and remaining water) of the extraction solutions were concentrated under reduced pressure at 45 °C and dessicated in vacuum. Through this method, the petroleum ether fraction (PF, 11.74 g), ethyl acetate fraction (EF, 66.67 g), and water fraction (WF, 225.34 g) were obtained. Also, XYXD extract and EF were dissolved in 0.3% sodium carboxymethyl cellulose (CMC-Na) solution for the *in vivo* experiment. PF, EF, and WF were dissolved in dimethylsulphoxide (DMSO) for the *in vitro* experiment, and dosages of PF, EF, and WF were converted into crude drug by yield extraction *in vitro* cell experiment, respectively.

Chemical analysis of XYXD extract and its fractions were analysed using UHPLC-Q-Orbitrap HRMS (Thermo Fisher Scientific Inc., Grand Island, NY, USA). UHPLC was Thermo Scientific Dionex Ultimate 3000 and controlled by Chromeleon 7.2 Software. The cooling autosampler was set at 10 °C and protected from light, and the column heater was set at 40 °C. Waters ACQUITY UPLC HSS T3 column (2.1 mm × 100 mm, 1.8 μm) was employed and the column temperature was set at 40 °C. The mobile phase consisted of A (0.1% formic acid) and B (acetonitrile) at a flow rate of 0.4 mL/min and eluted with gradient elution: 0–2 min (2% B), 2–12 min (2%–90% B), 12–17 min (90% B), 17–19 min (2% B). The injection volume was 5 μL. Mass spectrometer Q-Orbitrap system was connected to the UHPLC system via a heated electrospray ionisation and controlled by Xcalibur 4.1 software for data capture and analysis. The electrospray ionisation source was operated and optimised in negative ionisation mode. The optimised parameters of mass spectrometry were as follows: capillary temperature: 325 °C; sheath gas (N_2_) flow rate: 45 arbitrary units; auxiliary gas (N_2_) flow rate: 8 arbitrary units; sweep gas flow rate: 0 arbitrary units; spray voltage: 2.8 kV (negative); S-lens RF level: 50 V; auxilliary gas heater temperature, 300 °C; scan mode: full MS; scan range: 100–1500 *m/z*; maximum injection time (IT): 200 ms; scan resolution, 70,000 FWHM (*m/z*/s); and automatic gain control (AGC) target: 1.0 e6.

### Experimental animals and drug administration

Male C57BL/6J mice (SPF grade, weight of 20 ± 2 g and age of 6 weeks) and Sprague-Dawley (SD) rats (male, SPF grade, weight of 200 ± 20 g and age of 6 weeks) were supplied by the Shanghai Experimental Animal Centre of the Chinese Academy of Sciences and were fed in Experimental Animal Centre of Shanghai University of Traditional Chinese Medicine. All of animals were housed in an environment-controlled breeding room at 25 °C with a 12 h dark/light cycle. All experiments performed in mice were in accordance with the State Committee of Science and Technology of P.R. of China on November 14th, 1988, and this study protocol was approved and monitored by the Animal Ethics Committee of Shanghai University of Traditional Chinese Medicine (PZSHUTCM190322008; Approval date: 13 April, 2018). Hepatic fibrosis was produced in C57BL/6J mice by intraperitoneal administration of 10% CCl_4_ diluted in olive oil in doses of 2 mL/kg body weight three-times weekly for six weeks. After treatment with CCl_4_ for three weeks, CCl_4_-treated mice randomly fall into 3 groups: CCl_4_ fibrosis model group, XYXD treatment group, and EF treatment group. From weeks 4–6, the mice received treatment with 0.3% CMC-Na or with XYXD (993 mg/kg, the dose was used human equivalent dose [60 kg, 13 g recipe per day]) or EF (213 mg/kg, the dose was converted by the extraction yield and also used human equivalent dose [60 kg, 13 g recipe per day]), respectively, by gavage per day. Furthermore, the normal control mice were intraperitoneal injected olive oil at a concentration of 2 mL/kg body weight and were given 0.3% CMC-Na orally. At the end of the sixth week, all mice were sacrificed by anaesthesia, liver tissues and blood samples were collected for follow-up studies by histopathological, immunofluorescent assay, serum biochemistry, etc.

### Effect of different fractions on primary HSCs activation

Primary HSCs were isolated from male SD rats. Briefly, rats were cut open through the stomach, thereby fully exposing the liver after anaesthetisation with 3% sodium pentobarbital. The liver was sequentially perfused *in situ* through portal vein with 50 mL perfusion fluid, pronase E (1 mg/mL), and collagenase D (0.7 mg/mL) solutions. Liver was removed and further digested *in vitro* with collagenase D (0.34 mg/mL), pronase E (0.34 mg/mL), and DNAse I (0.02 mg/mL). After 15 min, the liver tissue was filtered by a 100 µm-mesh. Cells were separated through Nycodenz gradient centrifugation as described previously (Zhang et al. 2014). Isolated HSCs were seeded in 30 mm Petri dishes (5 × 10^5^ cells/dishes) and were maintained in Dulbecco’s modified Eagle’s medium (DMEM) supplemented with 20% foetal bovine serum (FBS) and 1% double antibody (1% penicillin–streptomycin solutions) in humidified air at 37 °C with 5% CO_2_ (Thermo). After 7 days, cells were treated with 10 μM SB431542, 50 μg/mL PF, EF, and WF for 24 h, respectively. Cells were assigned to the following five groups: normal control, SB431542 positive control, PF, EF, and WF groups. Protein expressions of α-SMA in the cells were measured by Western blot assay.

### Cells lines culture and treatment

JS1 and LX2 cells were seeded in 12-well plates or 30 mm Petri dishes, and were maintained in DMEM supplemented with 10% FBS and 1% double antibody in at 37 °C with 5% CO_2_, respectively, HHSECs were seeded in 30 mm petri dishes and were cultured in endothelial cell growth medium supplemented with 5% FBS and 1% endothelial cell growth supplement and 1% double antibody in a similar cell culture environment. The cells were treated with different concentrations of drugs. JS1 and LX2 cells were assigned to these groups: normal control group, TGF-β1 group (5 ng/mL), TGF-β1 (5 ng/mL) + SB431542 positive control group, TGF-β1 (5 ng/mL) + PF group, TGF-β1 + EF group, TGF-β1 (5 ng/mL) + WF group; JS1 cells were also divided into these groups: normal control group, TGF-β1 group, TGF-β1 + 13 compounds from EF at a range of concentrations, or TGF-β1 (5 ng/mL) + Emo group, TGF-β1 (5 ng/mL) + Rhe group, TGF-β1 (5 ng/mL) + Alo group, TGF-β1 (5 ng/mL) + Pru group;. The mRNA expression levels of α-SMA and COL-1 in cells were measured by RT-PCR assay. The protein expression levels of α-SMA, COL-1, Bax, Bcl-2, cytochrome C, cleaved-caspase-3, caspase-3, p-ERK1/2, ERK, p-p38, p-38, p-JNK, and JNK in the cells were measured by Western blot assay.

### CCK-8 cell viability assay

Cell viability was conducted using CCK-8 assay according to the manufacturer’s instructions, with cells (JS1, LX2, L02, or HHSECs) seeded at 5000 cells/100 µL/well into 96-well plates and incubated for 24 h at 37 °C in 5% CO_2_, in which JS1 and LX2 cells were incubated with PF, EF, WF, or JS1 cells were incubated with 13 compounds of EF, whereas L02 and HHSECs were also incubated with EF for another 24 h in similar culture condition. Two h before the scheduled detection time, CCK-8 solution (10 μL) was added to each well, and cells were incubated for another 2 h. Then, absorbance at 450 nm was detected by microplate reader (BioTek, Hercules, CA, USA). Mean optical density values from four wells for each experiment served as the index of cell viability, and the experiments were repeated thrice. Cell viability rate (%) = (absorbance of drugs wells − absorbance of blank wells)/(absorbance of control wells − absorbance of blank wells) × 100.

### EdU cell proliferation assay

To analyse the cellular proliferation, EdU staining was conducted by BeyoClick™ EdU Cell Proliferation Kit with Alexa Fluor 594 according to the manufacturer’s instructions. Cells seeded in 12-well plates were incubated with EF (0, 12.5, 25 and 50 μg/mL) for 24 h, and then EdU was added into the medium until the final concentration was 10 μM with incubated for 2 h. After the incubation, the cells were washed with PBS to remove the medium and the free EdU probe. The cells were then fixed in 4% paraformaldehyde and were permeabilized with 0.3% Triton X-100 at room temperature for 15 min, respectively. The cells were incubated with Click reaction solution for 30 min before being stained with Hoechst for 10 min. After an additional wash with PBS, the cells were observed under an inverted fluorescence microscope DP80 (Olympus, Tokyo, Japan).

### Cell cycle analysis

Cell cycle analysis was performed by flow cytometry using the Cell Cycle and Apoptosis Analysis kit and the data was analysed using Flowjo. Briefly, JS1 and LX2 cells were cultured under the treatment of EF (0, 12.5, 25 and 50 μg/mL) for 24 h, and then harvested and fixed in cold 70% ethanol for 24 h, followed by staining with PI for 10 min at room temperature. The samples were examined by flow cytometry (Beckman-Coulter, CA).

### LDH cytotoxicity assay

LDH assay was assessed using LDH-cytotoxicity assay kit according to the manufacturer’s instructions. Briefly, JS1 and LX2 cells were seeded at 8000 cells/200 µL/well into 96-well plates and incubated with different concentrations of EF (12.5, 25, 50, 100, and 200 μg/mL) for 24 h, respectively. Following treatment, the cell culture plate was centrifuged using centrifuge at 400 *g* for 5 min. Then, 120 μL supernatant per well was transferred to another 96-well plate, and 60 μL solution from LDH assay kit was added and incubated for 30 min in the dark at room temperature. Finally, cell culture plates were read at an absorbance of 450 nm by microplate reader. Experiments were repeated in triplicate. Cell survival rate (%) = 100 − (absorption of treated wells − absorption of control wells)/(absorption of maximum cell enzyme activity wells − absorption of control wells) × 100.

### Serum biochemistry and hepatic Hyp content assays

AST, ALT and TBil, and hepatic Hyp contents were measured according to the manufacturer’s instructions. Hyp content was utilised to indirectly reflect collagen content of liver tissue, and was expressed as μg/g of wet weight.

### Histopathological and immunofluorescent assay

Liver specimens were fixed in 10% formalin solution, dehydrated in automated vacuum tissue processor, embedded in paraffin, sliced at 4 μm thickness, and stained H&E and Sirius red according to manufacturer’s instructions, respectively, which was used to estimate histopathological features and fibrosis stage. Furthermore, Sirius red-positive area was calculated, which could also indirectly reflect collagen content of liver tissue.

Liver tissues were embedded in optimal cutting temperature (OCT), frozen-sectioned at 8 μm thickness, and were fixed in cold acetone for 10 min. The specimens were incubated with respective primary antibodies, including antibodies against CD31 (1:100), VEGF (1:200), and Desmin (1:100), respectively. Subsequently, the fluorescent secondary antibodies (1:1000), and DAPI (1:1000) staining. Finally, these specimens were scanned by a confocal laser scanning microscope FV10i (Olympus, Tokyo, Japan), and representative images were displayed.

### Scanning electron microscopy (SEM) and transmission electron microscope (TEM) for LSECs defenestration

The mice were anaesthetized with 3% sodium pentobarbital and the abdominal cavity was opened by the end of treatment. Phosphate buffer solution (PBS) was douched and, subsequently, 2.5% gluteraldehyde was fixed in liver through the portal vein, and the livers were removed and cut into roughly 3 mm thickness in similar parts using PBS douching and 1% osmic acid fixing. Finally, the sections were scanned by SEM (Philips XL-30, Eindhoven, Netherlands) and TEM (Philips Tecnai-12 BioTmin, Eindhoven, Netherlands).

### Western blot assay

Liver tissues or cells were lysed using lysis buffer and protein concentrations were detected via BCA assay. Equal amounts of 25 µg loading protein samples were separated on SDS-PAGE, and were transferred to PVDF membranes. The membranes were blocked and incubated with respective primary antibody, such as rabbit monoclonal antibodies against α-SMA (1:1000), COL-1 (1:1000), Bax (1:1000), Bcl-2 (1:1000), cytochrome C (1:2000), caspase-3 (1:500), cleaved-caspase-3 (1:500), p-ERK 1/2 (1:1000), ERK (1:1000), p-p38 (1:1000), p-38 (1:1000), p-JNK (1:1000), and JNK (1:1000) overnight at 4 °C, and subsequently incubated with an IRDye 800 CW-coupled secondary antibody (1:10000). Protein bands were scanned and analysed using odyssey infra-red imaging system (LI-COR, Biosciences, UK).

### Real-time PCR

Total RNA was extracted from JS1 and LX2 cells with Trizol Reagents, and the extracted total RNA was reverse-transcribed using reverse transcriptase kits according to the manufacturer’s instructions. Relative expression levels of α-SMA and COL-1 mRNA were measured by ABI ViiA7 sequence detector (Applied Biosystems, CA), and results were analysed via ΔC*_T_* method. Primers sequences used for qPCR were as follows: Mouse α-SMA, forward 5′-CTGACAGAGGCACCACTGAA-3′ and reverse 5′-CATCTCCAGAGTCCAGCACA-3′; Mouse COL-1, forward 5′-TGACTGGAAGAGCGGAGAGT-3′ and reverse 5′-GACGGCTGAGTAGGGAACAC-3′; Mouse GAPDH, forward 5′-CTTTGGCATTGTGGAAGGGCTC-3′ and reverse 5′-GCAGGGATGATGTTCTGGGCAG-3′; Human α-SMA, forward 5′-TTGGCTTGGCTTGTCAGG-3′ and reverse 5′-GCTTTAGGGTCGCTGGAG-3′; Human COL-1, forward 5′-CGGAGGTATGCAGACAACGA-3′ and reverse 5′-ACGGGGCTGGCTTCTTAAAT-3′; Human GAPDH, forward 5′-AGCCACATCGCTCAGACACC-3′ and reverse 5′-GTACTCAGCGCCAGCATCG-3′.

### Statistical analysis

The significance values were calculated using one-way ANOVA followed *post hoc* LSD multiple comparisons procedure by SPSS 20.0 software. Statistical significance was considered at *P*<0.05 and data were displayed as means ± standard deviation (SD).

## Results

### Chemical analysis of XYXD and its fractions by UHPLC-Q-Orbitrap HRMS

**Figure 1.**
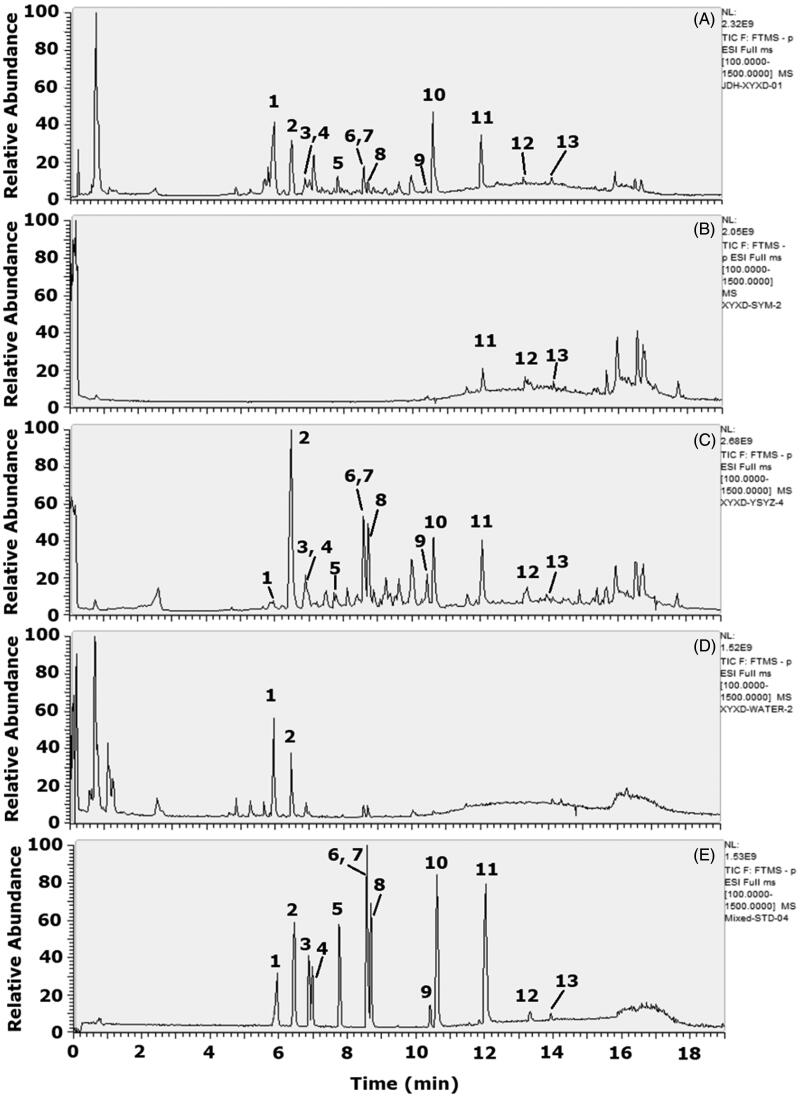
The typical fingerprint chromatograms of different fractions from XYXD. (A) The extract of XYXD. (B) The petroleum ether fraction (PF). (C) Ethyl acetate fraction (EF). (D) Water fraction (WF). (E) Reference standards. Amygdalin (Amy, **1**), prunasin (Pru, **2**), aloe-emodin-8-*O*-β-d-glucopyranoside (A-8-G, **3**), rhein-8-*O*-β-d-glucopyranoside (R-8-G, **4**), emodin-1-*O*-glucoside (E-1-G, **5**), emodin-8-glucoside (E-8-G, **6**), chrysophanol-8-*O*-β-d-glucopyranoside (C-8-G, **7**), chrysophanol-1-*O*-β-d-glucopyranoside (C-1-G, **8**), aloe-emodin (Alo, **9**), rhein (Rhe, **10**), emodin (Emo, **11**), chrysophanol (Chr, **12**) and physcion (Phy, **13**).

Petroleum ether fraction (PF, 11.74 g, mass transfer rate was 3.79%), ethyl acetate fraction (EF, 66.67 g, mass transfer rate was 21.51%), and water fraction (WF, 225.34 g, mass transfer rate was 72.69%) were obtained from the total extract of XYXD (310 g) using liquid–liquid extraction method. Typical fingerprint chromatograms of different fractions of the total extract, PF, EF, WF from XYXD, and mixture reference standards of Amy, Pru, A-8-G, R-8-G, E-1-G, E-8-G, C-8-G, C-1-G, Alo, Rhe, Emo, Chr, and Phy were deposited by UHPLC-Q-Orbitrap HRMS (Figure 1). The total extract of XYXD is rich in chemical components, and these components have been successfully transferred to different fractions according to different chemical properties of them. The contents of targeted markers Amy, Pru, A-8-G, R-8-G, E-1-G, E-8-G, C-8-G, C-1-G, Alo, Rhe, Emo, Chr, and Phy in the total extract of XYXD were determined as 18.78, 4.20, 0.6, 1.96, 0.13, 0.35, 0.25, 0.14, 1.36, 1.49, 1.87, 9.01, and 2.14 mg/g (Figure 1(A)), respectively. The contents of Emo, Chr, and Phy were 0.84, 2.67 and 0.93 mg/g in PF (Figure 1(B)). The contents of Amy, Pru, A-8-G, R-8-G, E-1-G, E-8-G, C-8-G, C-1-G, Alo, Rhe, Emo, Chr, and Phy were 76.24, 17.01, 2.64, 9.06, 0.53, 1.60, 1.03, 0.78, 6.19, 6.82, 5.56, 31.05, and 6.38 mg/g in EF (Figure 1(C)), respectively. The contents of Amy and Pru were 50.21 and 13.74 mg/g in WF (Figure 1(D)).

### EF distinctly suppresses proliferation and induces cell-cycle arrest of HSCs with no cytotoxicity

**Figure 2.**
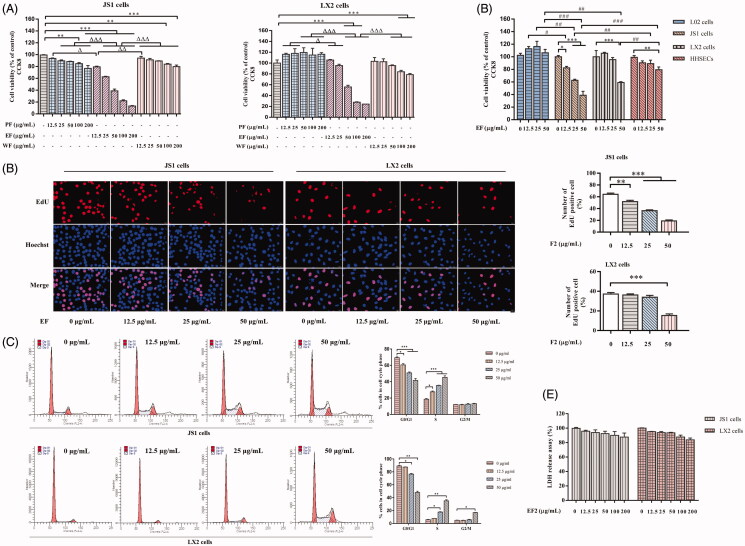
The effect of XYXD fractions on JS1 and LX2 cell viability, proliferating, cell cycle and cytotoxicity. (A) Cell viability of different fractions of XYXD on JS1 and LX2 cells. (B) Cell viability of EF on JS1 cells, LX2 cells, L02 cells and HHSECs. (C) The proliferation of EF on JS1 and LX2 cells, and the number of EdU-positive cells. (D) Cell cycle of EF on JS1 and LX2 cells. (E) Cytotoxicity of EF in JS1 and LX2 cells. The data are given in the form are of mean ± SD of three independent experiments. *p < 0.01, **p < 0.01, ***p < 0.001 versus control group (0.1% DMSO); ^Δ^*p* < 0.05, ^ΔΔ^*p* < 0.01, ^ΔΔΔ^*p* < 0.001 versus different fractions with the same dose. ^#^*p* < 0.05, ^##^*p* < 0.01, ^###^*p* < 0.001 versus different cells with the same dose. PF, the petrol ether extract of XYXD; EF, the ethyl acetate extract of XYXD; WF, the water extract of XYXD.

Different concentrations of PF, EF, and WF (12.5, 25, 50, 100, 200 μg/mL) were used in JS1 and LX2 cells for 24 h to investigate the proliferative effect of XYXD fractions against HSCs. CCK8 assay results indicate that EF showed evident inhibitory effect on the proliferation of JS1 and LX2 cells (Figure 2(A)). Further, the anti-proliferative effect of EF in different types liver cells, including L02 cells, JS1 cells, LX2 cells and HHSECs were comparatively studied, as shown in Figure 2(B). The inhibitory effect of EF was highly apparent in JS1 and LX2 cells compared with other cells, thereby indicating that EF selectively inhibits HSCs proliferation in liver cells. Nextly, the anti-proliferative effect of EF in JS1 and LX2 cells were further confirmed by EdU staining, suggesting that EF could significantly inhibit the proliferation of HSCs (*p* < 0.01 or *p* < 0.001) (Figure 2(C)). Meanwhile, the cell-cycle distributions were detected by flow cytometry, the results showed a decrease in the G0/G1 phase population and an increase in the S phase population in EF-treated JS1 and LX2 cells, as well as the G2/M phase population was increase in EF-treated LX2 cells with 50 μg/mL, compared with the no treatment group (*p* < 0.05 or *p* < 0.01 or *p* < 0.001), indicating EF induced cell cycle arrest at S phase or/and G2/M phase (Figure 2(D)). In addition, cytotoxicity of EF in JS1 and LX2 cells were also evaluated by LDH release assay (Figure 2(E)), and EF does not display significant cytotoxicity in HSCs. In short, these results demonstrated that EF could suppress the proliferation of HSCs without apparent cytotoxicity, possibly by increasing the percentage of cells in the S phase or/and G2/M phase.

### EF remarkably suppresses HSCs activation

**Figure 3.**
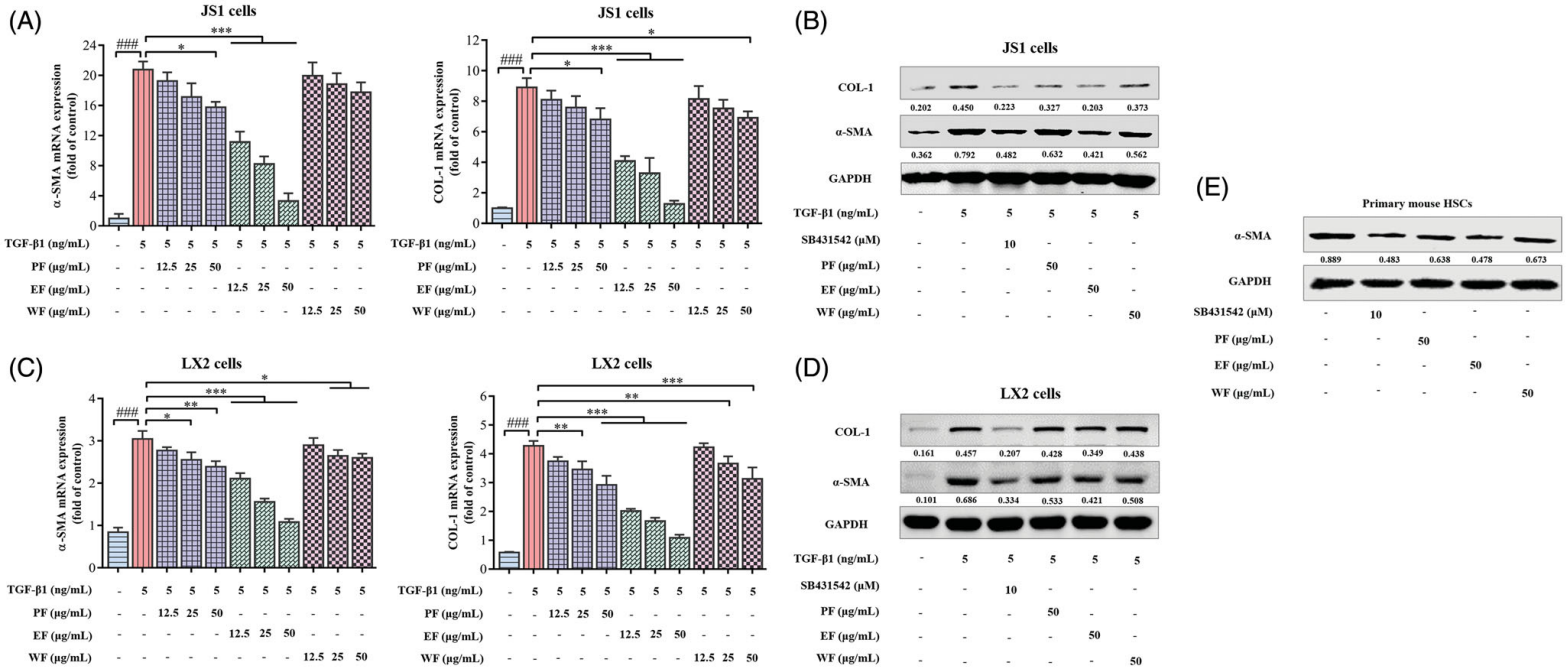
The effect of different active fractions of XYXD on activation of HSCs. (A) The mRNA expression of α-SMA and COL-1 in JS1 cells. (B) The protein expression of α-SMA and COL-1 in JS1 cells. (C) The mRNA expression of α-SMA and COL-1 in LX2 cells. (D) The protein expression of α-SMA and COL-1 in LX2 cells. (E) The protein expression of α-SMA in primary mouse HSCs. The data are expressed as the mean ± SD of three independent experiments. ^###^*p* < 0.001 versus control group (0.1% DMSO). **p < 0.05, **p < 0.01, ***p < 0.001 versus model group (5 ng/mL TGF-β1). PF, the petrol ether extract of XYXD; EF, the ethyl acetate extract of XYXD; WF, the water extract of XYXD.

Fractions were used in TGF-β1-induced activation of HSCs or self-activation of primary HSCs to evaluate the inhibitory effect of different fraction extracts of XYXD for HSCs activation. As expected, mRNA expression levels of α-SMA and COL-1 in JS1 cells were remarkably reduced in treatment with EF (12.5, 25, and 50 μg/mL), and its effects were mostly distinct, whereas in treatment with PF and WF, apparent effects were only at a concentration of 50 μg/mL (Figure 3(A)). Further, protein expression levels of α-SMA and COL-1 in JS1 cells were also assessed at 50 μg/mL of PF, EF, and WF. Western blot results showed that the suppression effects of EF in JS1 cell was similar to SB431542 (TGF-β inhibitor), and its effect was highly robust than other fractions (Figure 3(B)). Notably, consistent results were achieved in LX2 cells and primary HSCs (Figure 3(C–E)). EF remarkably inhibited the activation of HSCs with a superior effect than other fraction extracts. EF was implied as the main active fraction of XYXD on HSCs activation.

### EF ameliorates liver injury and hepatic fibrogenesis in mice induced by CCl_4_

**Figure 4.**
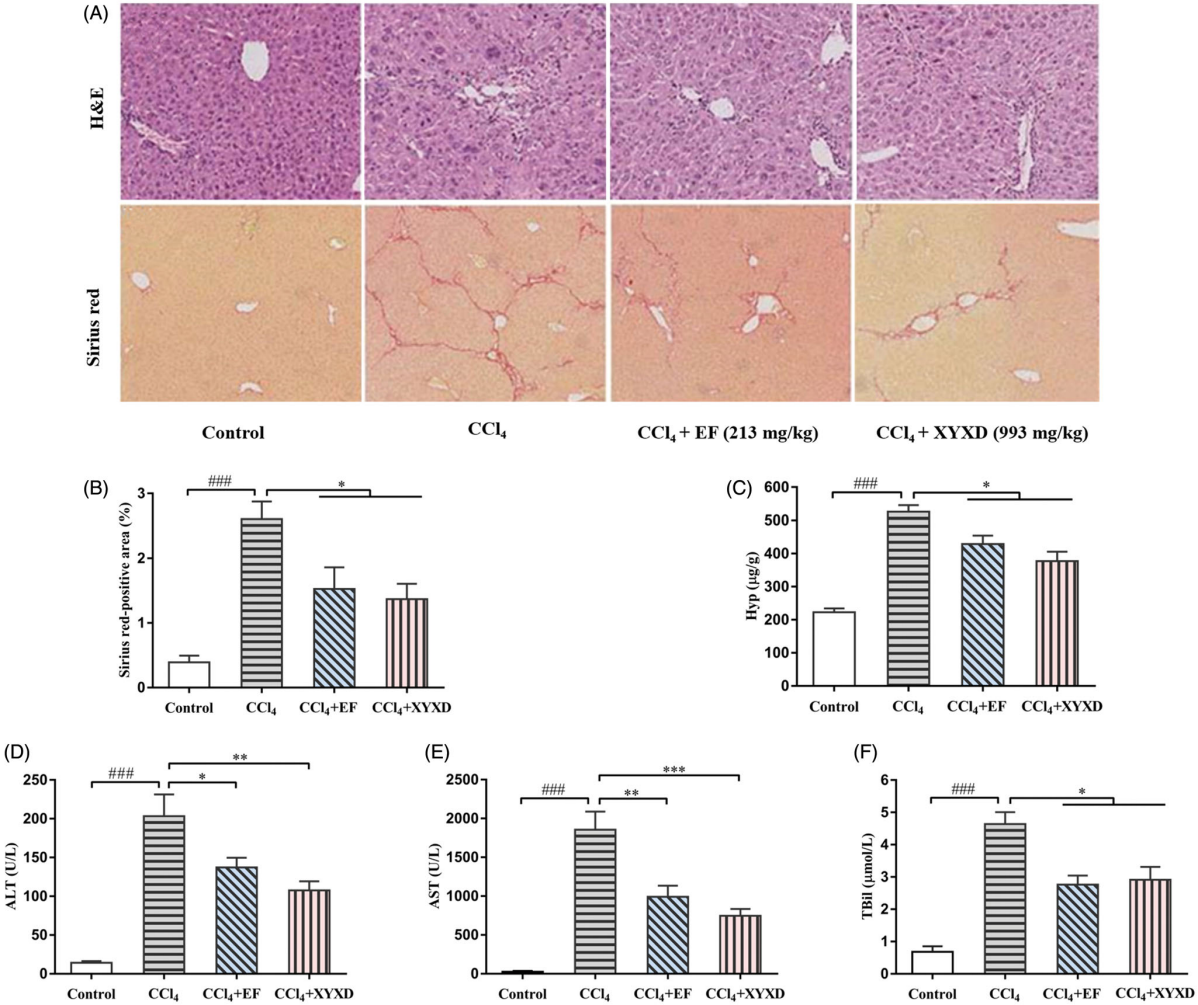
The effect of EF on liver injury and fibrosis in mice indued by CCl_4_. (A) H&E (×200) staining and Sirius red (×100) staining. (B) Sirius red-positive area. (C) Hyp content. (D–F) Serum levels of ALT, AST, and TBil. The results are expressed as the mean ± SD. ^###^*p* < 0.001 versus control group. *p < 0.05, **p < 0.01, ***p < 0.001 versus CCl_4_ model group. ALT: alanine aminotransferase; AST: aspartate aminotransferase; TBil: total bilirubin.

On the basis of inhibitory effect of EF on HSCs proliferation and activation *in vitro*, exploring the therapeutic effect of EF on hepatic fibrosis *in vivo* is interesting. As shown in Figure 4(A), the changes of liver histopathology in mice by H&E staining, identified that the morphological abnormality, such as sinusoidal dilatation, hepatocyte steatosis, inflammation and inflammatory cells infiltration around the central venous, causes liver damage induced by CCl_4_ in varying degrees. However, EF-treated mice clearly improve these changes highly similar with XYXD-treated mice, which suggest that EF ameliorates pathologic changes in liver damage. Similarly, the indexes of liver function, such as the serum levels of ALT, AST, and TBil, were also assessed in these mice. As shown in Figure 4(D–F), ALT, AST, and TBil levels were remarkably increased in CCl_4_-induced mice compared with the control mice (*p* < 0.01). However, the treatment with EF had a beneficial effect on liver function. The serum levels of ALT, AST, and TBil were distinctly improved similar with XYXD compared to the CCl_4_-induced mice (*p* < 0.05 or *p* < 0.01). EF effectively improved liver function in liver injury.

Furthermore, the degree of liver fibrosis was evaluated by Sirius red staining, several fibrous septum formations, collagen fibrous proliferation, and deposition, were observed in the fibrotic liver tissues, and the fibrosis was identified as stage 3 (Figure 4(A)). As with XYXD-treated mice, the collagen depositions were reduced, fibrous septa were not pronounced, and fibrotic stages were excessively reduced in EF-treated mice. EF ameliorated fibrosis progression in mice induced by CCl_4_.

In addition, Sirius red-positive area, an indirect indicator of collagen content in the liver tissue, was calculated. Statistics showed that Sirius red-positive area was extensively reduced in EF treatment group compared with the CCl_4_ treatment group (*p* < 0.01, Figure 4(B)), which approximated to XYXD treatment group. The liver tissue Hyp content, an important indicator for collagen metabolism, was measured simultaneously to further verify collagen deposition in liver fibrosis. As shown in Figure 4(C), the liver Hyp content in the CCl_4_ group was more than twice the control group (*p* < 0.01), which implies excessive deposition of collagen in the liver fibrotic tissues. However, a distinct reduction of the liver Hyp content was observed in EF-treated mice similar with XYXD-treated mice (*p* < 0.05). These data indicate that EF reduces collagen deposition during liver fibrosis. Overall, data suggest that EF significantly inhibits the development of hepatic fibrosis induced by CCl_4_, and its effect closely approximates XYXD.

### EF drives HSCs apoptosis in CCl_4_-induced fibrosis

**Figure 5.**
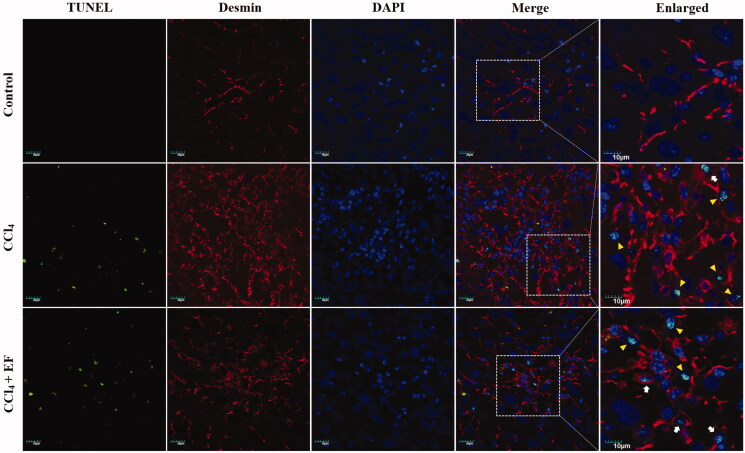
The effect EF on HSCs apoptosis in fibrotic liver tissue. Immunofluorescence staining of TUNEL and Desmin. The cells nuclei were counterstained with DAPI. Scale bar = 20 µm and 10 µm. The white arrow shows the cellular distribution of TUNEL and Desmin in HSCs, the yellow triangles shows the cellular distribution of TUNEL in other intrahepatic cells (excluding HSCs). Representative confocal microscopy images are shown. DAPI: 4′,6-diamidino-2-phenylindole; TUNEL: TdT-mediated dUTP nick-end labelling.

On the basis of good treatment effects of EF for liver fibrosis, exploration of the anti-hepatic fibrosis mechanisms of EF is essential. Inducing HSCs apoptosis is an effective method to treat liver fibrosis (Tsuchida and Friedman 2017). Thus, apoptosis of HSCs in liver tissues was investigated using immunofluorescent assay. As shown in Figure 5, some scattered green (TUNEL, apoptotic marker) or red granules (Desmin, HSCs marker) appeared in the liver tissues of EF-treated mice and CCl_4_ group mice; whereas, dual immunofluorescence staining displayed more co-localization of TUNEL and Desmin in the liver tissues of EF-treated mice compared with CCl_4_ group mice. Apparently, EF induced the apoptosis of HSCs in CCl_4_-induced fibrosis.

### EF regulates the expression level of mitochondrial apoptotic pathway-related proteins *in vitro*

**Figure 6.**
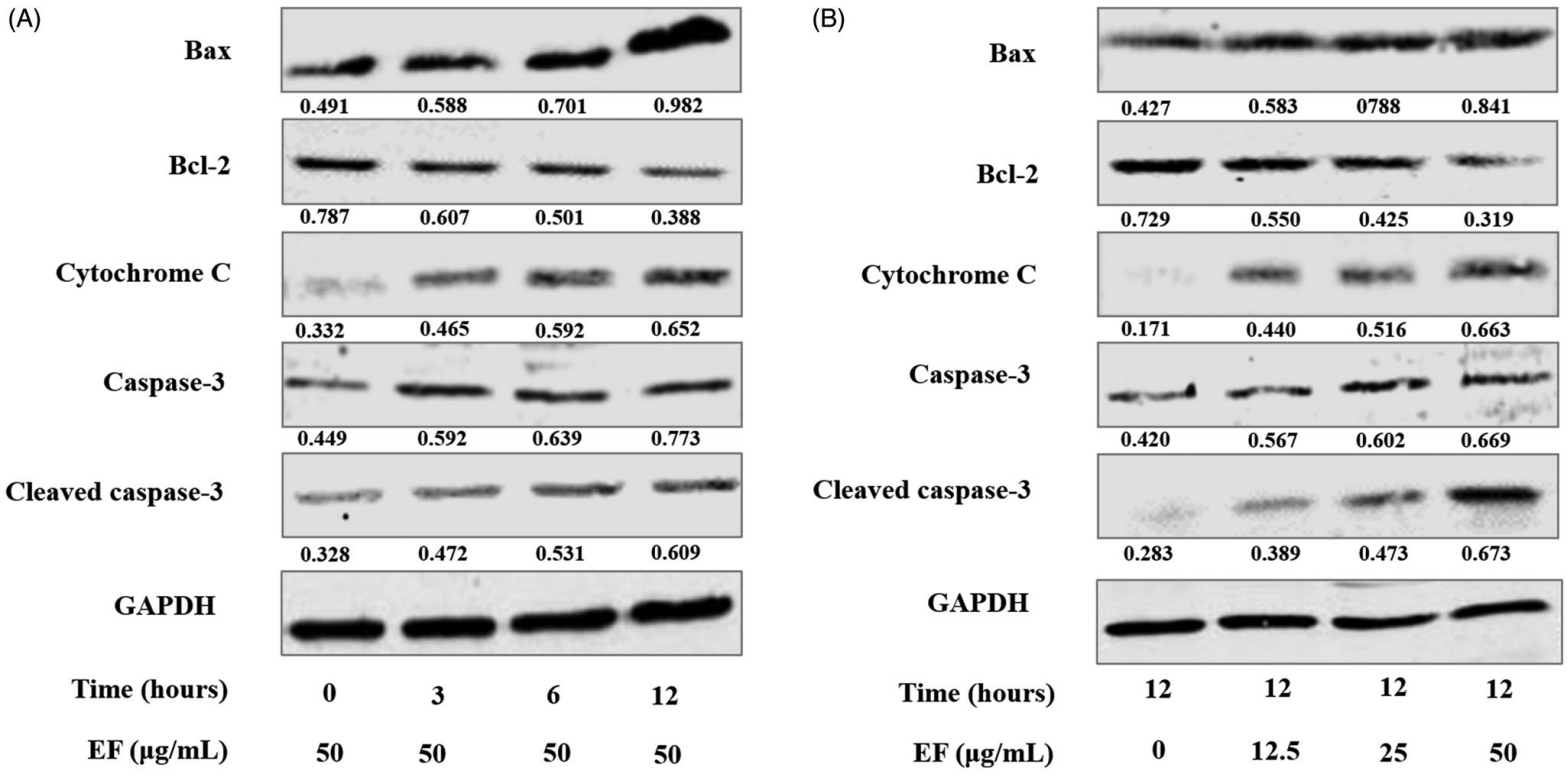
EF regulates the expression level of mitochondrial apoptotic pathway-related proteins in JS1 cells. (A) The expression of mitochondrial apoptotic proteins of EF (50 μg/mL) in JS1 cells with different times. (B) The expression of mitochondrial apoptotic proteins of EF at a range of concentrations for 12 h. Similar results were observed in three independent experiments.

Mitochondria play a crucial effect in cellular apoptosis. Hence, relative apoptotic protein changes of mitochondrial apoptosis pathway were further investigated in JS1 cells with different times and doses of EF. Cells were treated for 3, 6, and 12 h, respectively. Western blot assay shows that protein expression levels of Bax, cytochrome C and caspase-3 and cleaved-caspase-3 were clearly upregulated in response to the EF treatment (50 μg/mL) with prolonged incubation time (Figure 6(A)). Bax initiated cell apoptosis by forming various sizes of pores in mitochondrial outer membrane that led to the release of cytochrome C into the cytoplasm, thereby subsequently accelerating the activation of the pro-apoptotic caspase cascade, such as cleavage of caspase-3. Bcl-2 can restrain the release of cytochrome C (Aouacheria et al. 2007). Notably, expression levels of Bcl-2 were clearly reduced. Additionally, in terms of dose, 12.5, 25, and 50 μg/mL of EF were used in the cells for 12 h. As shown in Figure 6(B), these proteins were significantly regulated. Particularly, EF regulated pro- and anti-apoptotic proteins expression in HSCs. EF regulated the expression of mitochondrial apoptotic proteins in HSCs. EF induced HSCs apoptosis possibly via the mitochondrial apoptotic pathway.

### EF selectively activates JNK and p38 mitogen-activated protein kinases (MAPKs) signalling pathways

**Figure 7.**
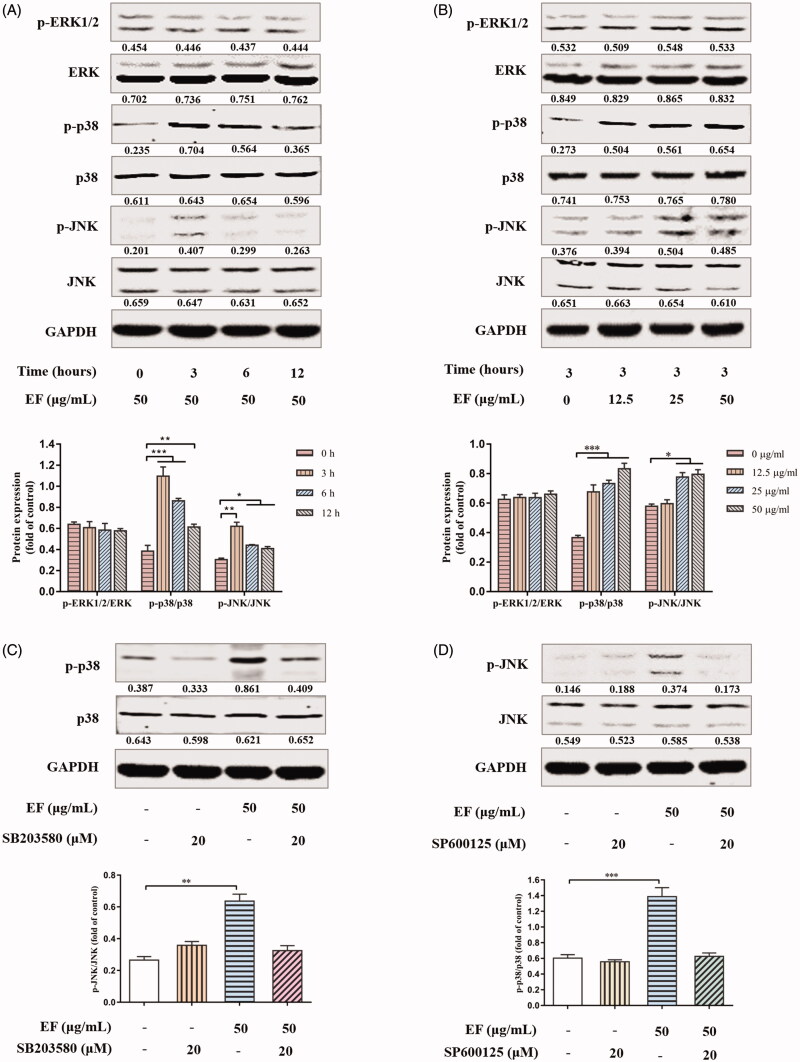
EF regulates JNK and p38-MAPKs signalling pathways in JS1 cells. (A) The proteins expression of phosphorylated and total ERK1/2, p38 and JNK in JS1 cells with 50 μg/mL EF under different times. (B) The proteins expression of phosphorylated and total ERK1/2, p38 and JNK in JS1 cells with EF at a range of concentrations for 3 h. (C) The proteins expression of phosphorylated and total p38 in JS1 cells. (D) The proteins expression of phosphorylated and total JNK in JS1 cells. Similar results were observed in three independent experiments. *p < 0.05, **p < 0.01, ***p < 0.001 versus control group (0.1% DMSO).

MAPKs, as the upstream regulator of the mitochondrial apoptotic pathway, indicate participation in cellular apoptosis according to accumulating evidence (Kohsuke et al. 2011). Therefore, ERK1/2, JNK, and p38 MAPKs were observed in JS1 cells at a specific time and concentration of EF. As shown in Figure 7(A,B), EF selectively promoted JNK and p38 phosphorylation but not ERK1/2 phosphorylation. Interestingly, the peak decreased gradually after 3 h. SP600125 (JNK inhibitor) and SB203580 (p38 inhibitor) were used in JS1 cells to further confirm selectivity of EF for JNK and p38, and inhibitors neutralised the influences of EF for JNK and p38 (Figure 7(C,D)). Therefore, JNK and p38 activation may be vital for EF-induced HSCs apoptosis in mitochondrial apoptotic pathway.

### EF suppresses LSECs defenestration and angiogenesis

**Figure 8.**
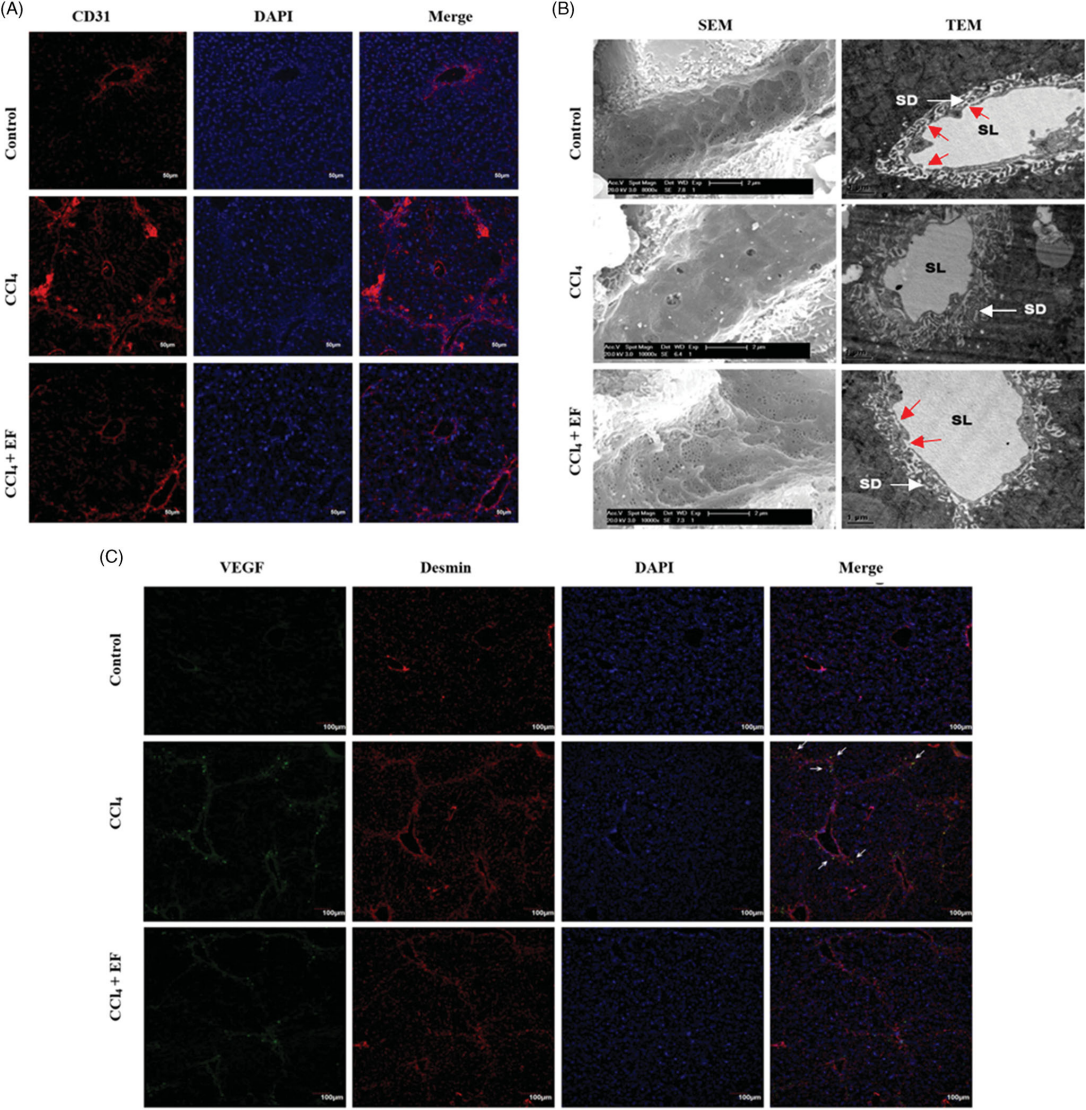
The effect of EF on LSECs defenestration and angiogenesis. (A) Immunofluorescence staining of CD31 in fibrotic liver, the nuclei were counter stained with DAPI, representative confocal microscopy images are shown, scale bar = 50 µm. (B) SEM (left) and TEM (right), the white arrows represent space of Disse, the red arrows represent endothelial fenestration of pores, representative images are shown. (C) Immunofluorescence staining of VEGF and Desmin in fibrotic liver, the nuclei were counterstained with DAPI, Representative confocal microscopy images are shown, Scale bar = 100 µm. The white arrows represent co-localization of VEGF and Desmin. SEM: scanning electron microscope; SEM: scanning electron microscope; SD: space of Disse; SL: sinusoid lumen; DAPI: 4′,6-diamidino-2-phenylindole.

LSECs also play an important role in hepatic fibrosis. Thus, LSECs defenestration was also evaluated in this study. First, CD31 (a marker of the dedifferentiated, nonfenestrated LSECs) was observed in liver tissues by immunofluorescent staining of CD31. As shown in the Figure 8(A), we observed overexpression of CD31 in large vessel zones, such as portal and central veins, the hepatic sinusoidal wall, fibrous septum, and portal area in the fibrotic liver tissues relative to the normal liver tissues. However, overexpression in the liver tissue was clearly lowered by EF treatment. Therefore, EF suppressed the defenestration of LSECs during liver fibrosis.

SEM and TEM were used to observe changes of LSECs ultrastructure estimating LSECs defenestration in fibrotic liver; SEM assay showed that LSECs have abundant small open fenestrae in sieve plates and lack a basement membrane in the normal liver tissues. In contrast, the fibrotic liver tissues displayed LSECs lack endothelial fenestration of pores on the surface of sinusoids, and only a small number of open fenestrae in sieve plates. Remarkably, EF improves these changes of the liver tissue of CCl_4_ model mice (Figure 8(B), left). As expected, similar results were also observed in TEM assay (Figure 8(B), right). In normal liver tissues, the sinusoidal cavity was round and uncompressed. LSECs have several open fenestrae in sieve plates that lack basement membrane and diaphragm. After treatment with CCl_4_, liver tissues showed several hyperplasia in the space of Disse, and large amount of fenestration and pores or gapsdisappeared, and basement membranes formed in the sinusoid lumen. However, changes of LSECs in the fibrotic liver tissues were improved by EF treatment. Thus, results suggested that EF ameliorated LSECs defenestration in CCl_4_-induced fibrosis.

VEGF, a key regulator of LSECs phenotype and growth factor of angiogenesis, was also studied. As shown in the Figure 8(C), an abundant co-localization of VEGF and Desmin in the portal area were observed in fibrotic liver tissues relative to the normal liver tissues, whereas co-localization was reduced in EF-treated liver tissues. Thus, EF inhibited the expression of VEGF in HSCs *in vivo*. Together, these results suggested that EF could repress LSECs destruction and angiogenesis, and regulate the expression of VEGF secreted by HSCs.

### Effects of main chemical ingredients of EF on activation of HSCs

**Figure 9.**
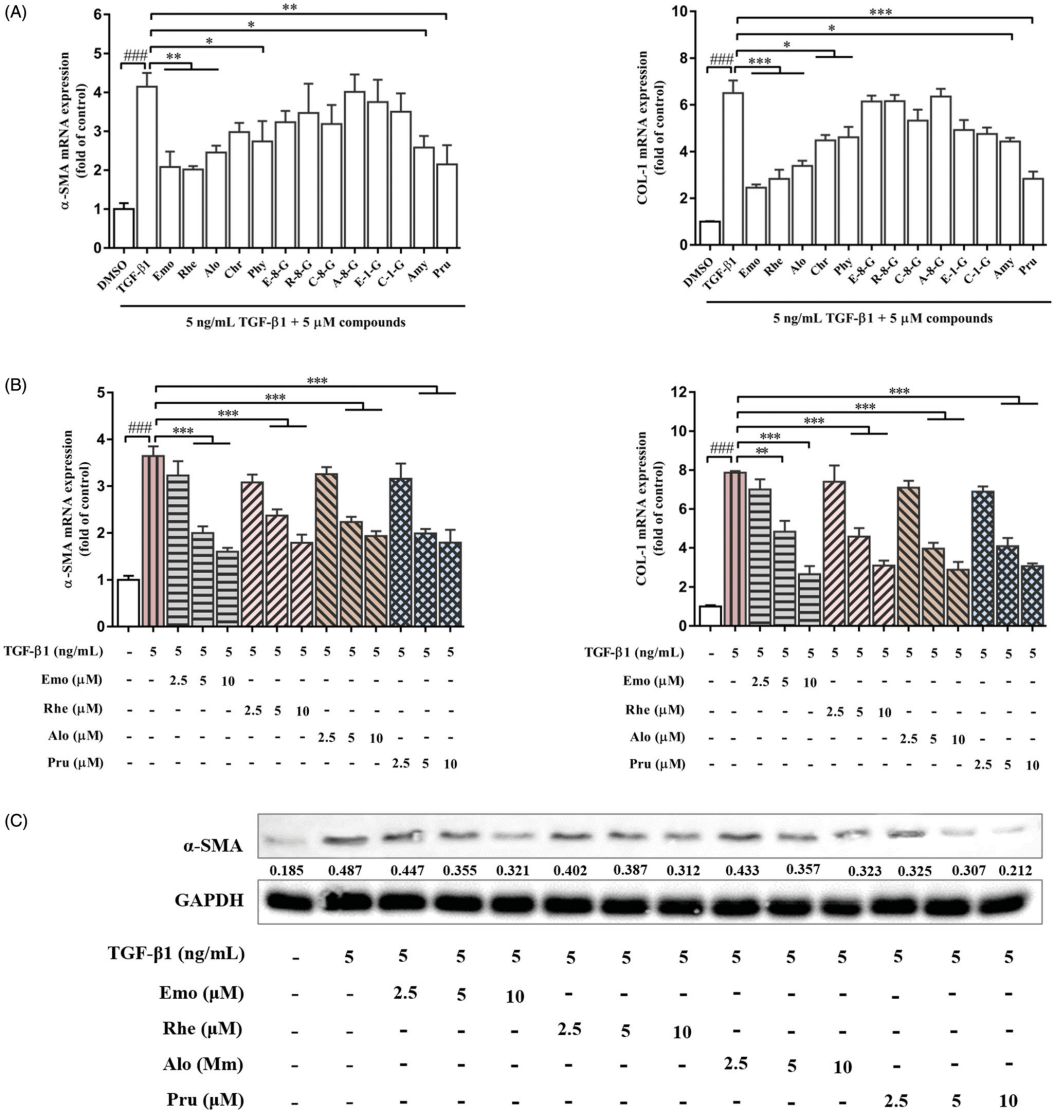
The effects of 13 compounds in EF on JS1 cells. (A) The mRNA expression of α-SMA and COL-1 in JS1 cells with 13 compounds at concentration of 5 μM. (B) The mRNA expression of α-SMA and COL-1 in JS1 cells with emodin, rhein, aloe-emodin and prunasin at concentration of 2.5, 5, 10 μM. (C) The protein expression of α-SMA in JS1 cells with these four compounds at concentration of 2.5, 5, 10 μM. ^###^*p* < 0.001 versus control group (0.1% DMSO); *p < 0.05, **p < 0.01, ***p < 0.001 versus model group (5 ng/mL TGF-β1). Emo: emodin; Rhe: rhein; Alo: aloe-emodin; Chr: chrysophanol; Phy: physcion; E-8-G: emodin-8-glucoside; R-8-G: rhein-8-*O*-β-d-glucopyranoside; C-8-G: chrysophanol-8-*O*-β-d-glucopyranoside; A-8-G: aloe-emodin-8-*O*-β-d-glucopyranoside; E-1-G: emodin-1-*O*-glucoside; C-1-G: chrysophanol-1-*O*-β-d-glucopyranoside; Amy: amygdalin; Pru: prunasin.

On the basis of anti-hepatic fibrosis effect of EF and the analytic results of UHPLC-Q-Orbitrap HRMS, the effects of 13 compounds of EF on HSCs were evaluated. First, the mRNA expression level of α-SMA and COL-1 in JS1 cells were evaluated when these ingredients at 5 μM. The results show that the mRNA expression level of α-SMA and COL-1 of Emo, Rhe, Alo, Chr, Phy, Amy, and Pru were suppressed, except for the expression of α-SMA of Chr, in which the suppression effects of Emo, Rhe, Alo, and Pru were highly prominent (Figure 9(A)). Moreover, these four compounds with different concentrations (2.5, 5, and 10 μM) were further evaluated in the cellular mRNA and protein expression of α-SMA and COL-1 (Figure 9(B,C)). Together, these results indicated that these 13 compounds may be potential active ingredients of EF that exert anti-hepatic fibrosis effects, especially Emo, Rhe, Alo, and Pru, but they still require further study.

## Discussion

TCM has accumulated rich clinical experiences for centuries in many diseases including chronic liver diseases. Recently, the effect of herbal medicines and Chinese compound formula on liver fibrosis has been gaining much attention from many researchers (Dong et al. 2018; Eissa et al. 2018). XYXD, as one of classical prescription in TCM, is usually used to treat chronic liver diseases, such as fibrosis and cirrhosis, except for gynecological diseases in China. Hepatic fibrosis is caused by the activation of HSCs and the excessive accumulation of ECM protein (Tsuchida and Friedman 2017). XYXD could suppress the activation of HSCs and deposition of collagens *in vitro* and *in vivo* (Zhang et al. 2014; Liu et al. 2015). In addition, XYXD could also alleviate destruction of LSECs and apoptosis of intestinal epithelial cells in CCl_4_-induced liver fibrosis (Zhang et al. 2014; Ma et al. 2017). However, its mechanism and active ingredients of anti-hepatic fibrosis of XYXD still remain unclear. In the present study, the inhibitory effects of different fraction extracts for liver fibrosis were evaluated, especially the main active fraction EF, as well as the underlying mechanism of anti-fibrosis and main ingredients of EF.

Based on the effective anti-hepatic fibrosis effect of XYXD in CCl_4_-induced liver fibrosis from our previous studies (Zhang et al. 2014), clarifying its underlying mechanisms and active ingredients is essential. The proliferation and activation of HSCs are closely related to the occurrence and progression of liver fibrosis (Schuppan and Kim 2013). In this study, EF is the main active fraction of XYXD, which could clearly suppress cells proliferation, induce cells cycle arrest in the S phase, and inhibit cells activation with no distinct cytotoxicity in HSCs, and the possible anti-proliferative effect of EF on HSCs was superior to hepatocytes and LSECs. Cell cycle is an ordered set of highly regulated events that allow cell to grow, duplicate its genetic material, and divide into two daughter cells, which accomplished through specific cyclins that act in association with cyclin-dependent kinases. Cell cycle arrest is a stopping point in the cell cycle, where it is no longer participated in the processes of duplication and division (Poon 2016). Therefore, EF-mediated the inhibition of cell proliferation may be induced by suppressing DNA synthesis through inducing S-phase cell cycle arrest. Interestingly, EF did not show obvious cytotoxicity in inhibiting cell proliferation, we speculate that EF-mediated cell cycle arrest may also induce apoptosis. Importantly, apoptosis is important for the elimination of activated HSCs, which will be investigated later in the mechanism study. Furthermore, EF was further used to evaluate its anti-fibrosis effect in fibrotic mice, and results from our experiment showed apparent improvement of liver function and histology changes, distinct reduction of fibrotic degree were observed in EF-treated mice that was highly similar with XYXD-treated mice. EF was the main active fraction of XYXD with many compounds and showed significant suppression effect during hepatic fibrosis *in vitro* and *in vivo*. The different fraction extracts were analysed using UHPLC-Q-Orbitrap HRMS, which identified 13 targeted markers Amy, Pru, A-8-G, R-8-G, E-1-G, E-8-G, C-8-G, C-1-G, Alo, Rhe, Emo, Chr, and Phy in XYXD and EF, but rarely identified the ingredients in PF and WF. As far as we know, PF might contain a large number of fat-soluble components such as fats, sphingosine, and volatile oils. WF might contain large amounts of polysaccharides, nucleosides, water-soluble amino acids and other water-soluble ingredients. Most of these ingredients are difficult to analyse by UHPLC-Q-Orbitrap HRMS. The effects of main chemical ingredients of EF on activation of HSCs was found that the apparent inhibition of mRNA expression level of α-SMA and COL-1 in most components, which implies that these compounds were potential active ingredients. Among them, the effect of Emo, Rhe, Alo, and Pru was particularly prominent. Interestingly, some previous studies have confirmed that Emo, Rhe, Alo, Chr, Phy, and Amy have shown different degrees of anti-liver fibrosis effects in *in vivo* and *in vitro* experiments (Woo et al. 2002; Lian et al. 2005; Lin et al. 2008; Li et al. 2016; Lian et al. 2017; Wang et al. 2018).

HSCs apoptosis is essential for resolution of liver fibrosis, as it can reduce the number of activated HSCs (Schuppan and Kim 2013). Importantly, the involvement of mitochondria in apoptosis is one of the most important pathways, which through mitochondrial permeability transition pore-opening release of cytochrome C into the cytoplasm from mitochondria to activate caspases. Bax is considered as the promoter, and conversely, Bcl-2 is the inhibitor of cell apoptosisthus, Bax initiates cell apoptosis, but Bcl-2 inhibits cell apoptosis (Aouacheria et al. 2007). In the present study, immunofluorescent assay displayed a co-localization of TUNEL and Desmin in EF-treated fibrotic tissues, thereby implying that EF accelerates apoptosis of HSCs. Subsequently, up-regulated expression levels of Bax, cytochrome C, caspase-3, and cleavage of caspase-3 proteins, and down-regulated expression of Bcl-2 proteins were observed in EF-treated JS1 cells. EF induced HSCs apoptosis possibly by regulating the expression of apoptotic proteins in the mitochondrial apoptosis pathway. Previous studies showed that JNK and p38, as the upstream signal regulators of the mitochondrial apoptotic pathway, play an important role in apoptotic processes of different cell types. Activated JNK and p38 may affect the expressions of apoptotic genes and/or proteins, including pro- and anti-apoptotic Bcl-2 family member through transcriptional and post transcriptional mechanisms, thus, facilitating induction of apoptosis, i.e., through JNK activation, can repress the anti-apoptotic activity of Bcl-2 via phosphorylation (Bogoyevitch and Kobe 2006; Dhanasekaran and Reddy 2008; Wagner and Nebreda 2009). Pro-and anti-apoptotic Bcl-2 family proteins can also promote JNK-induced apoptosis pathways (Kohsuke et al. 2011). In the present study, JNK and p38 were distinctly activated in EF-treated HSCs, and their activations were neutralised in EF-treated HSCs pre-treated with JNK inhibitor and p38 inhibitor, which suggested that EF selectively activated JNK and p38. Collectively, these results indicated that anti-fibrotic mechanism of EF may be related to induction of HSCs apoptosis. EF activated Bax and suppressed Bcl-2, and subsequently many sizes of pores formation in mitochondrial outer membrane, which accelerated the release of cytochrome C into the cytoplasm and facilitated caspase-3 activation and cleavage, thereby initiating apoptosis. The process may be mediated by EF-activated JNK and p38 MAPKs, but further research is needed.

Capillarization is a change from LSEC phenotype to vascular phenotype, which occurs before liver fibrogenesis; Capillarization permits the activation of HSCs, thereby further accelerating fibrogenesis (DeLeve et al. 2008a, 2008b). Remarkably, defenestration is a trait of capillarization of LSECs (Xie et al. 2012). CD31, as a marker of LSECs dedifferentiation, is usually measured to evaluate the change of LSECs phenotype (DeLeve et al. 2004). Furthermore, activated HSCs can secrete some molecules such as VEGF and angiopoietin-1, which promote angiogenesis of LSECs (Thabut and Shah 2010). VEGF is an important growth factor of angiogenesis and a critical regulator of LSECs phenotype (DeLeve 2015). In the present article, LSECs lacked endothelial fenestration of pores on the surface of sinusoids, and only a small amount of open fenestra was present in sieve plates in fibrotic tissue from morphologic fields by SEM and TEM analysis, whereas abundant expressions of CD31 and VEGF in fibrosis tissue were observed. Clearly, EF could ameliorate these changes. The co-localization of VEGF and Desmin was observed by immunofluorescent assay in fibrotic liver, thereby suggesting that EF suppressed LSECs defenestration and angiogenesis probably by regulating the expression of VEGF secreted by HSCs during fibrosis.

## Conclusions

EF had apparent suppressing effect on HSCs proliferation and activation *in vitro* and exhibited decent therapeutic effects similar to those of XYXD on CCl_4_-induced liver fibrosis. Results implied that EF was the main active fraction extracts of XYXD. Furthermore, results further demonstrated that the anti-fibrotic mechanism of EF may be related to the induction of HSCs apoptosis through regulating mitochondrial apoptotic pathway by selectively activating the JNK and p38-MAPKs signalling pathways. Moreover, EF showed significant suppression effect on LSECs dedifferentiation during hepatic fibrosis. In addition, the potential active ingredients of EF anti-hepatic fibrosis may be connected with its various compounds, especially Emo, Rhe, Alo, and Pru. Results can be confirmed and extended to future studies.
